# IDO1 promotes *Echinococcus multilocularis* infection by regulating the formation of neutrophil extracellular traps

**DOI:** 10.1186/s13567-025-01572-2

**Published:** 2025-07-01

**Authors:** Haining Zhang, Ru Meng, Fan Zhang, Ao Chen, Hongrun Ge, Wangkai Chen, Zhi Li, Yong Fu

**Affiliations:** 1 Academy of Animal Sciences and Veterinary Medicine, Qinghai Provincial Key Laboratory of Pathogen Diagnosis for Animal Diseases and Green Technical Research for Prevention and Control, Qinghai University, Xining, 810016 China; 2 State Key Laboratory for Diagnosis and Treatment of Severe Zoonotic Infectious Diseases, Key Laboratory for Zoonosis Research of the Ministry of Education, Qinghai University, Xining, 810016 China; 3 Xining Animal Disease Control Center, Xining, 810016 China

**Keywords:** *Echinococcus multilocularis*, neutrophil extracellular traps, IDO1, NF-κB

## Abstract

The widespread prevalence of alveolar echinococcosis (AE) caused by *Echinococcus multilocularis* infection poses a significant threat to human health. *E. multilocularis* is found primarily in the Northern Hemisphere. Given the limitations of current treatment methods, primarily surgical resection, there is a pressing need for more effective therapeutic options. We established a mouse model of *E. multilocularis* infection by injecting *E. multilocularis* protoscoleces into C57BL/6 mice. The formation of neutrophil extracellular traps (NETs) following *E. multilocularis* infection was identified and validated using various techniques, including transcriptome sequencing, scanning electron microscopy (SEM) and flow cytometry. We found that the knockout of the Indoleamine 2, 3 dioxygenase 1 (IDO1) gene or the administration of IDO1 inhibitors resulted in a decrease in the levels of NET-related molecules, including *CitH3*, *MPO*, *PAD4*, *PR3*, *NE*, and MPO–DNA complexes, as well as cfDNA. In addition, after the addition of the IDO1 inhibitor, the levels of p65, phospho-p65, p50/105, and REL increased. These results showed that IDO1 promotes the formation of NETs and inhibits NF-κB activation. Moreover, IDO1 inhibits AE progression by regulating NET formation. In conclusion, this study revealed that IDO1 inhibits AE progression by regulating NET formation, and this regulation may be associated with IDO1-induced neutrophil production and NF-κB signalling activation. These results are valuable for understanding the pathogenesis of *E. multilocularis* and may offer new insights for the prevention and treatment of AE.

## Introduction

Echinococcosis is a zoonotic parasitic disease caused by the larvae of *Echinococcus* spp. [1]. The widespread prevalence of alveolar echinococcosis (AE) in the Northern Hemisphere, caused by *E. multilocularis* infection, poses a significant threat to human health [2]. After invading the human body, the eggs hatch in the intestines and then migrate primarily to the liver, where they settle and gradually develop into metacestodes (larval stage) in a tumour-like manner [3]. Currently, the mainstay of treatment for AE is surgical resection, but the success of surgery is influenced by several factors [4]. Therefore, a better understanding of the immune response to *E. multilocularis* may help in the development of effective treatment strategies.

Neutrophils, the most abundant immune cells in human blood, are a vital component of the human immune system [5]. They play a crucial role in defending against the invasion of pathogens, including bacteria, viruses, and parasites [6]. As a critical component of the first line of defence against microbial pathogens, neutrophils use several mechanisms to respond to pathogen invasion, including phagocytosis, degranulation and the formation of neutrophil extracellular traps (NETs) [7, 8]. NETs have attracted much attention as a newly discovered host defence mechanism. NETs are network structures composed of granular proteins, histones, and cytoplasmic proteins that can capture pathogens through their “stickiness” [9–11].

At present, research on NETs encompasses viruses [12], bacteria [9], fungi [13], and parasites. Regarding the role of NETs in parasitic infections, research has shown that infection with *Toxoplasma gondii* stimulates NET production by neutrophils in both humans and mice, thereby restricting the invasion of *T. gondii* [14]. The invasion of hookworms can also stimulate NET formation, but hookworms can degrade the DNA backbone by secreting deoxyribonucleases, which subsequently impacts NET formation [15]. The excretory/secretory products of *Trichinella spiralis* can inhibit NET formation by suppressing the production of reactive oxygen species (ROS) [16]. A study revealed a significant increase in the neutrophil-to-lymphocyte ratio after infection with *E. multilocularis* [17]; in addition, studies have shown that NETs form in patients and mice infected with *E. multilocularis* [18, 19]. However, the role of NETs in *E. multilocularis* infection has not been specifically studied.

Indoleamine 2, 3 dioxygenase 1 (IDO1) is a critical enzyme that regulates the catabolism of tryptophan by converting it into kynurenine, the core regulatory factor in immune suppression [20]. IDO1 can inhibit the proliferation of CD4 + and CD8 + T lymphocytes and natural killer (NK) cells [21]. Additionally, it can induce the activation of regulatory T cells (Tregs) and myeloid-derived suppressor cells (MDSCs) [22]. Studies have shown that IDO1 promotes AE progression by suppressing the function of T cells [3, 23]. However, the impact of IDO1 on NET formation and neutrophils during *E. multilocularis* infection has not been reported.

Nuclear factor kappa-B (NF-κB), a multifunctional transcription factor, plays crucial roles in inflammation, angiogenesis, and cell proliferation in tumours [24]. Research has shown that inhibiting the MyD88/NF-κB pathway can exacerbate liver injury and fibrosis in AE [25]; additionally, mmu-miR-374b-5p can regulate the expression of inflammatory factors during *E. multilocularis* infection by modulating the C/EBPβ/NF-κB signalling pathway in Kupffer cells [26]. NF-κB in cancer cells promotes angiogenesis by increasing the expression of C-X-C motif chemokine receptor 4 (CXCR4) and CXCL8/interleukin-8 (IL-8) [27]. IL8 is a protein that possesses neutrophil chemotactic properties [28]. In addition to regulating the recruitment and activation of neutrophils, IL-8 can also regulate the chemotaxis and activity of other white blood cells [29]. The increased formation of NETs in breast cancer may be related to the activation of the NF-κB pathway [30]; however, the role of the NF-kB pathway in NETs formed by *E. multilocularis* infection has not yet been studied.

In the present study, after *E. multilocularis* infection, the number of neutrophils increased. *E. multilocularis* stimulation induced the formation of NETs. Furthermore, IDO1 promoted the progression of AE, facilitated the production of neutrophils and contributed to the formation of NETs. We conclude that IDO1 promotes the progression of AE by regulating the formation of neutrophils and NETs.

## Materials and methods

### Animals

C57BL/6 mice and IDO1-deficient (IDO1^−/−^) C57BL/6 mice aged 8–10 weeks were purchased from Jiangsu GemPharmatech Co. Ltd. All the mice were housed in a specific pathogen-free environment with rodent chow and water ad libitum on a 12 h/12 h light–dark cycle. *E. multilocularis* protoscoleces (PSCs) were collected from the peritoneum of C57BL/6 mice with lesions and washed four times with phosphate-buffered saline (PBS, pH = 7.2, containing 1000 mg/mL penicillin and 1000 U/mL streptomycin) [31]. PSCs with viability above 95% were counted, and 2000 PSCs were injected intraperitoneally into each mouse [32].

### Blood collection and isolation of neutrophils

The mice were sacrificed after being infected for 3 months, and the liver and blood were collected in a sterile environment. Liver leukocytes were isolated as previously described [33]. In brief, the liver was cut into small pieces using surgical scissors, ground, and then passed through a 70 μm cell filter. The cell suspension was centrifuged at 500 × *g* for 5 min at 4 °C, after which the cells were resuspended in 37.5% Percoll (Biotopped, Beijing, China) and centrifuged at 1800 × *g* for 15 min at 23 °C. The supernatant was discarded, and 1 mL of ACK buffer (erythrocyte lysis buffer) was added and incubated at room temperature for 7 min. Then, 14 mL of cold RPMI 1640 medium was added, the mixture was centrifuged at 500 × *g* for 5 min at 4 °C, and the cells were resuspended in cold RPMI 1640 medium. Neutrophils were isolated using anti-Ly-6G magnetic beads following the manufacturer’s instructions (Miltenyi Biotec, Bergisch Gladbach, Germany).

### Flow cytometry

The isolated liver leukocytes were resuspended in staining buffer and incubated with TruStain FcX^™^ PLUS anti-mouse CD16/32 antibody (clone S17011E; Biolegend, USA) at 4 °C for 30 min. Then, the cell surface markers were stained with the mAbs APC anti-mouse Ly-6G, FITC anti-mouse/human CD11b, and PerCP/Cyanine5.5 anti-mouse CD45 (clones 1A8, M1/70, and 30-F11, respectively, Biolegend, USA) at 4 °C for 30 min. After the cells were washed twice with the staining buffer and then resuspended in the staining buffer, the cell phenotypes were detected via a BD FACSCelesta™ flow cytometer (BD Biosciences) and analysed with FlowJo V10 software.

### Immunofluorescence

Neutrophils were fixed with 4% paraformaldehyde, permeabilized with 0.1% Triton X-100, blocked with 5% BSA at room temperature, and then incubated overnight with anti-CitH3, anti-MPO, anti-PAD4, and anti-PR3 (ab281584 1:1000, ab270441 1:100, ab214810 1:1000, ab208670 1:100, respectively, Abcom, UK) and anti-NE (sc-55549, 1:100; Santa Cruz Biotechnology, Santa Cruz, USA) antibodies at 4 °C. Afterward, the sections were incubated with the corresponding Alexa Fluor™ 488/594-conjugated secondary antibodies (Thermo Fisher Scientific, USA) at room temperature for 1 h. All the samples were counterstained with DAPI (AR1176, BOSTER, Wuhan, China). Before each step, all the samples were washed three times with PBS. Finally, TissueFAXS Spectra Multispectral Cytometers (TissueGnostics, Austria) were used to observe the cells.

### Western blot

Liver tissue or cell protein was extracted using the T-PER™ Tissue Protein Extraction Reagent (Thermo Fisher Scientific, USA). Protease inhibitors and phosphatase inhibitors were added to the liver tissue, and then T-PER™ was used for homogenization. Then, SDS‒PAGE sample buffer was added, and the samples were boiled for sodium dodecyl sulfate‒polyacrylamide gel electrophoresis. The proteins were transferred to a polyvinylidene fluoride membrane (Merck Millipore, Germany) and blocked with 5% skim milk. The membrane was incubated overnight at 4 °C with anti-CitH3, anti-MPO, anti-PAD4, anti-PR3, anti-NFkB p65 (phospho S536), anti-NFkB p65, anti-NFkB p105/p50 (ab281584 1:1000, ab270441 1:1000, ab128086 1:2000, ab133613 1:1000, ab76302 1:1000, ab32536 1:1000, ab32360 1:1000, Abcam, UK), anti-NE (sc-55549, 1:200; Santa Cruz Biotechnology, USA) and anti-β-actin (66009-1-Ig, 1:50000; Proteintech Group, USA) antibodies. HRP-conjugated goat anti-mouse/rabbit IgG (ab6789, ab6721, Abcam, UK) was incubated with the membrane as the secondary antibody at room temperature for 1 h. An eBLOT Chemiluminescent western blot imager (Beijing Tronck Technology, China) was used to scan the membrane, and the image was quantified using ImageJ.

### RNA extraction and quantitative real-time PCR

Total RNA was extracted from liver tissue, cells or cysts with an RNeasy Mini Kit (Qiagen, Canada) according to the manufacturer’s instructions. cDNA was synthesized with the PrimeScript^™^ FAST RT Reagent Kit with gDNA Eraser (RR092A; Takara Biomedical Technology, China). The expression of *CitH3*, *PAD4*, *PR3*, *NE*, *MPO*, *14-3-3* and *II/3-10* was analysed using the TB Green^®^ Premix Ex Taq^™^ II FAST qPCR reagent (CN830A, Takara Biomedical Technology, China) with the primers listed in Table 1. A QuantStudio^™^ real-time fluorescence quantitative PCR system was used for detection, glyceraldehyde 3-phosphate dehydrogenase (*GAPDH*) or *β-actin* was used for normalization, and relative expression was quantified using the 2^−ΔΔCT^ method.

**Table 1:** **Primers used for q‒PCR**

Gene	Forward (5´-3´)	Reverse (5´-3´)
*Cit H3*	TCGACCGAGCTGCTGATCCG	TCAAACAGACCCACGAGGTAGGC
*PAD4*	GCTGGATGCCTTTGGGAACCTG	CGCTGCTGGAGTAACCGCTATTC
*PR3*	AGCAGCAGAAGTTCACCATCAGTC	AGCCACCGCCACCTCCTTG
*NE*	GTGCCGCCGTCGTGTGAAC	CCAAGGGTCCGCCAGAGTCC
*MPO*	ACGCCTGGAGTCAATCGCAATG	ACGCTCCTGGTCCTTGGTCAG
*REL*	GAGAAACCAAGAACTGCCCCTCTG	TGCCCTGGAACTCCTGAAGACC
*14–3-3*	GATAGTACTCTCATCATGCAG	CTCAATCAGAACCACGACAG
*II/3–10*	GGAGGAACGATTGCAACGTA	TCTTACTCTCATAGGCAGATG
*GAPDH*	CGGATTTGGTCGTATTGGG	CTCGCTCCTGGAAGATGG
*β-actin*	CGCGATCTCACCGACTGG	CTCCAGAGAGGAGCTAGTG

### Measurement of the MPO-DNA complex and cfDNA

The level of the MPO-DNA complex was detected as previously described [34]. In brief, the biotinylated mouse anti-MPO antibody (HM1051BT; Hycult Biotech, USA) was coated onto the coating layer of the ELISA plate of the Cell Death Detection ELISA Kit (Roche, Switzerland). The plate was incubated overnight at 4 °C. After washing with PBST three times, the plate was blocked with 1% BSA for 2 h. According to the manufacturer’s instructions, 20 µL of mouse serum was added to each well. The plate was then oscillated at 300 rpm for 2 h at room temperature. After washing three times with incubation buffer, the ABTS solution was added, and the mixture was incubated at 250 rpm for 10 min. The ABTS stop solution was then added, and the absorbance was measured via a BioTek Synergy H1 (BioTek, USA) at a wavelength of 405 nm.

In accordance with previous reports [35], cell-free DNA (cfDNA) was purified from mouse whole blood using the QIAamp DNA Mini Kit (QIAGEN, Germany) following the manufacturer's instructions. The concentration of cfDNA in whole-blood DNA was subsequently measured with a Quant-iT PicoGreen dsDNA quantification kit (Thermo Fisher Scientific, USA). Lambda DNA (Thermo Fisher Scientific, USA) was diluted with Tris–EDTA (TE) buffer (10 mM Tris–HCl, 1 mM EDTA, pH 7.5) to prepare standard DNA samples. TE buffer, whole blood samples, and Quant iT PicoGreen dsDNA reagent were added to the 96-well black microplate. The mixture was incubated at room temperature in the dark for 10 min, after which the fluorescence signal of the sample was measured via a Cytation 5 (BioTek, USA) at an excitation wavelength of 480 nm and an emission wavelength of 520 nm.

### In vitro experiments

As mentioned earlier, the isolated neutrophils were resuspended in 1640 medium and seeded onto a 6/12-well plate at a density of 1 × 10^6^ per well. After being cultured at 37 °C and 5% CO_2_ for 30 min, the cells were stimulated with 100 nmol/L PMA (P8139, Sigma, USA), cyst fluid, and PBS for 4 h; other groups of cells were stimulated with PBS, cyst fluid, an IDO1 inhibitor (B6036, APExBIO, USA), and an IL-8 inhibitor (SB225002, Selleck, USA) for 60 h each. The preparation process for cyst fluid was as follows: a sterile syringe was used to extract cyst fluid from *E. multilocularis*-infected mouse liver cysts. The sample was heated at 4 °C and 500 × *g* for 10 min to remove host cells and large fragments. The supernatant was collected and then filtered through a 0.22 μm membrane to eliminate bacteria and small particles. Finally, the filtered fluid was packaged into sterile cryovials for long-term storage at −80 °C.

For immunofluorescence, the cells were incubated with anti-CitH3 and anti-NE antibodies as described previously and then observed with a Nikon A1R laser confocal microscope (Nikon, Japan). For scanning electron microscopy, the cells were fixed overnight with 4% glutaraldehyde. After washing three times with PBS, gradient dehydration was performed using ethanol (30%, 50%, 70%, 80%, 90%, and 100%). After drying and spraying with gold, the samples were observed and photographed using a JSM-6610LV scanning electron microscope (JEOL, Japan).

### RNA sequencing and data analysis

Total RNA was extracted from cells using the RNeasy Mini Kit (Qiagen, Canada) according to the manufacturer’s instructions. The RNA libraries were sequenced on an Illumina HiSeq 2500 sequencing platform (Gene Denovo Biotechnology Co., Ltd., Guangzhou, China). The gene expression levels were calculated via the RPKM method. The “digital gene expression profile significance” algorithm was used to identify differentially expressed genes (DEGs) across each library, and *P*-values were used to detect DEGs that were statistically significant. A false discovery rate (FDR) of ≤ 0.001 and an absolute log_2_ ratio of ≥ 1 are established as thresholds to determine the significance of gene expression differences. The functions of the DEGs were annotated via gene set enrichment analysis (GSEA) and Kyoto Encyclopedia of Genes and Genomes (KEGG) pathway enrichment analysis (*P* ≤ 0.05).

### Statistical analysis

This was a single-blinded randomized controlled trial, and all the data are presented as the mean ± standard error of the mean (SEM). A t-test was used for statistical analysis. All analyses were conducted using GraphPad Prism 9.0, and values with *P* < 0.05 were considered statistically significant (*P < 0.05, **P < 0.01, ***P < 0.001).

## Results

### *E. multilocularis* induces NET formation, and IDO1 influences neutrophil production

**Figure 1:**
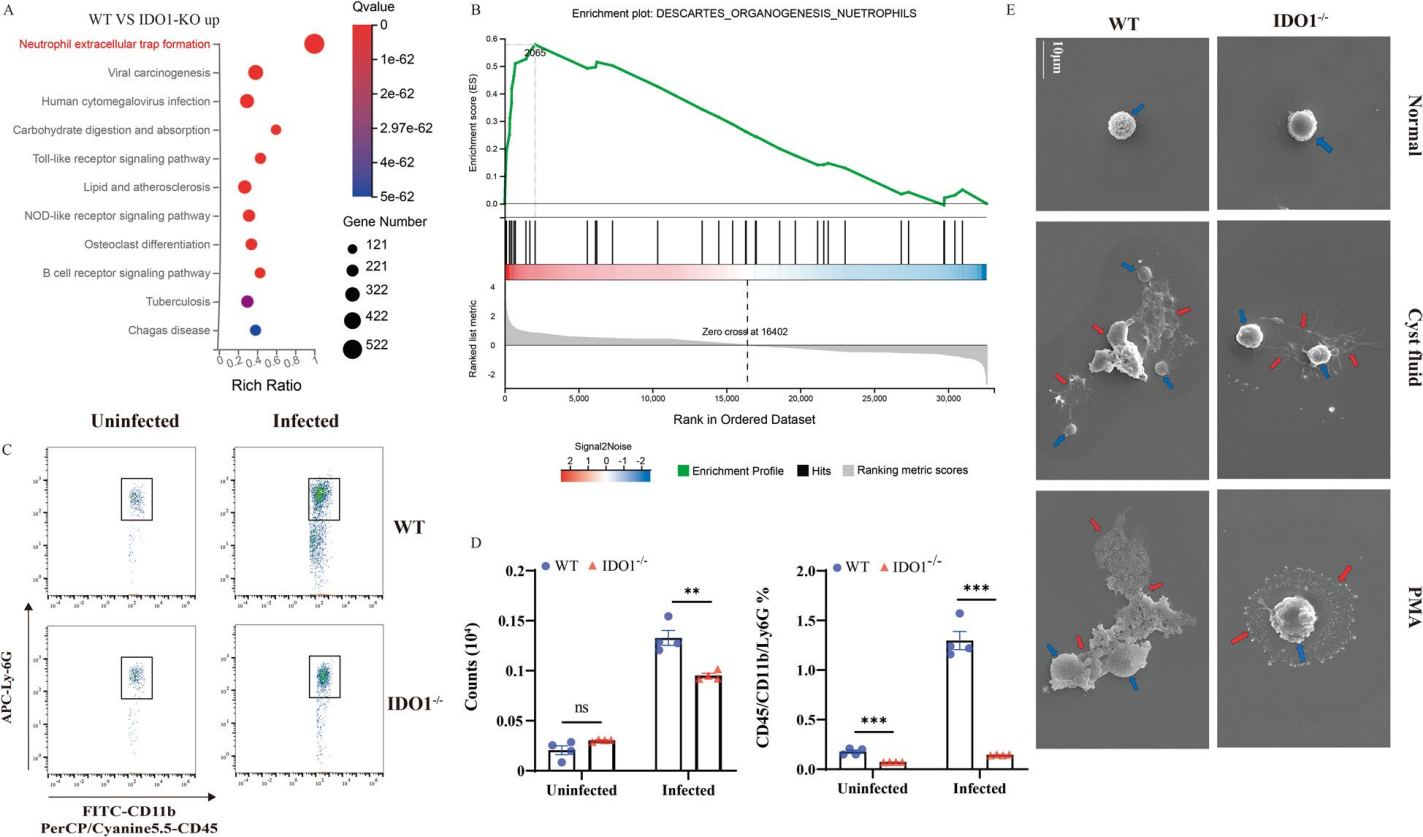
***E. multilocularis* can stimulate neutrophils to form NETs.** **A** KEGG pathway enrichment analysis was performed on differentially expressed genes. **B** Gene set enrichment analysis of neutrophils. **C** Flow cytometry analysis of uninfected and infected WT mice and IDO1-KO mice using Flowjo. **D** Number and percentage of CD45 + CD11b + Ly6G neutrophils. **E** Neutrophils stimulated with cyst fluid or PMA under SEM; the red arrow indicates NETs. The data represent the mean ± SEM; ns, not significant; *P < 0.05; **P < 0.01; ***P < 0.001.

KEGG pathway enrichment analyses of the DEGs revealed that the NET formation pathway was significantly enriched (Figure 1A). GSEA was used to predict the KEGG downstream pathway of IDO1. The results showed that IDO1 expression could activate Descartes organogenesis neutrophil signalling (Figure 1B). Neutrophils in mouse livers were quantified by flow cytometry, and the data revealed a significant increase in neutrophil infiltration in the livers of the mice after *E. multilocularis* infection. These results indicate that IDO1 can promote the generation of neutrophils. Additionally, WT mice presented greater liver neutrophil infiltration than IDO1-KO mice did (Figures 1C, D). After stimulating neutrophils isolated from WT-uninfected mice with cyst fluid in vitro, NETs formed (Figure 1E). These data indicate that *E. multilocularis* can induce the formation of NETs.

### IDO1 promotes the progression of AE and the formation of NETs

**Figure 2:**
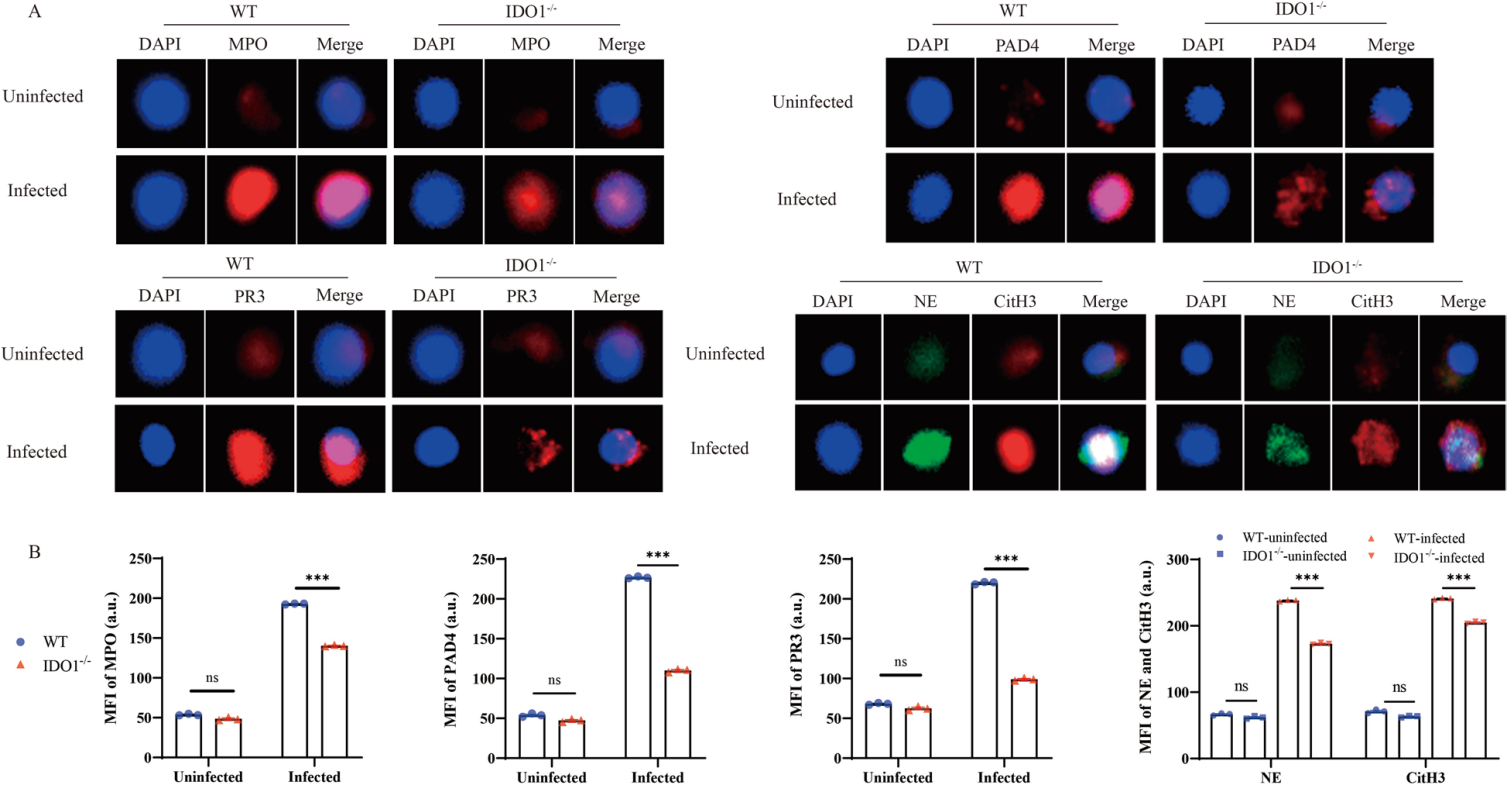
**Immunofluorescence detection of NET formation.** **A** Levels of NET-related molecules (*MPO*, *PAD4*, *PR3*, *NE* and *CitH3*) were visualized and evaluated by TissueFAXS Spectra Multispectral Cytometers, and areas of colocalization (merged) are shown. Blue: DAPI; **B** Bar chart of average fluorescence intensity. The data represent the mean ± SEM; ns, not significant; *P < 0.05; **P < 0.01; ***P < 0.001.

**Figure 3:**
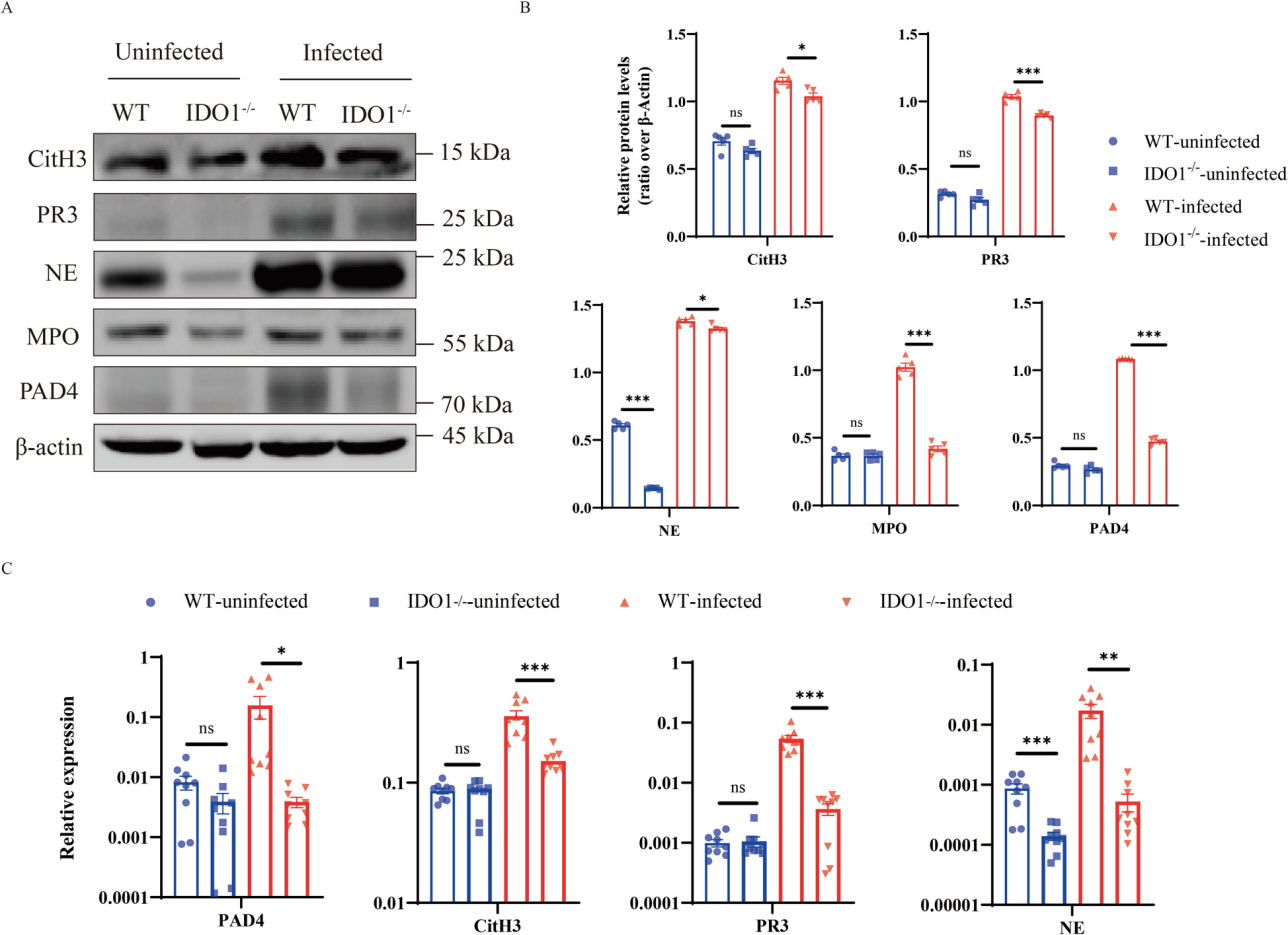
**IDO1 promotes the formation of NETs.** **A** Immunoblotting analysis of the expression of NET-related molecules (*MPO*, *PAD4*, *PR3*, *NE* and *CitH3*); **B** Bar chart for grayscale analysis via ImageJ software; mRNA transcription levels of *PAD4*, *PR3*, *NE* and *CitH3*. The data are presented relative to the GAPDH values and are presented as the mean ± SEM; ns, not significant; * *P* < 0.05; **P < 0.01; ***P < 0.001.

**Figure 4:**
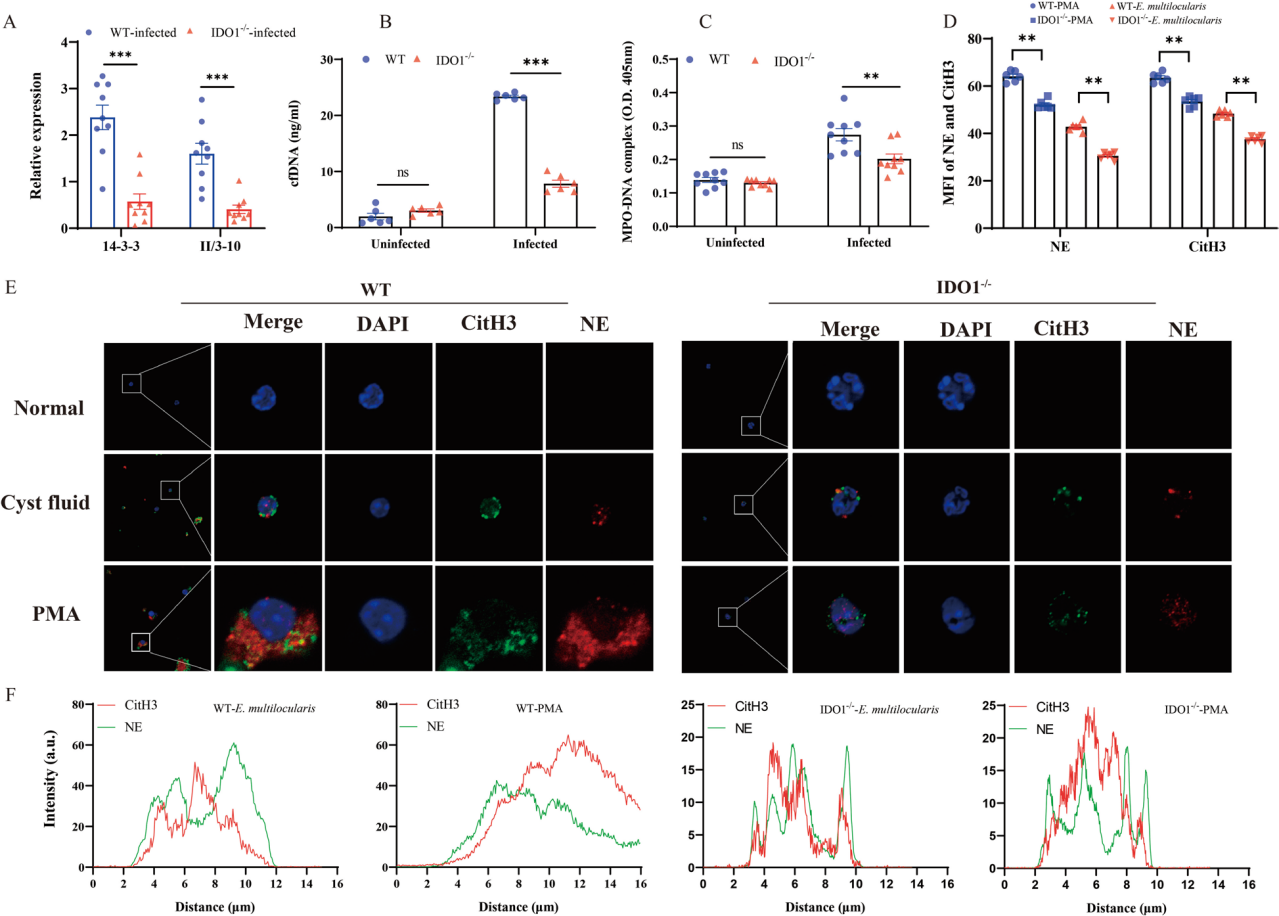
**IDO1 promotes the progression of AE and the formation of NETs in vitro A** mRNA transcription levels of the specific expression molecules *14–3-3* and *II/3–10* in *E. multilocularis*; **B** Detection of cfDNA levels in mouse whole blood; **C** Detection of MPO‒DNA complex levels in mouse serum. **D** Bar chart of average fluorescence intensity. **E** Levels of *NE* and *CitH3* expression were visualized and evaluated by confocal microscopy, and areas of colocalization (merges) are shown. **F** Plot profile tool was used to represent partial colocalization between *NE* and *CitH3*. Blue: DAPI; green: *CitH3*; red: *NE*. The data are presented relative to the GAPDH values and are presented as the mean ± SEM; *P < 0.05; **P < 0.01; ***P < 0.001.

To further investigate the impact of IDO1 on the formation of NETs, we analysed NET expression in mice before and after infection with *E. multilocularis* using various methods. Immunofluorescence analysis revealed a significant increase in the expression of *CitH3*, *NE*, *MPO*, *PAD4* and *PR3* after *E. multilocularis* infection. The expression was greater in WT mice than in IDO1-KO mice (Figures 2A, B). Afterward, we validated the immunofluorescence results using WB and qPCR, the results of which were consistent with previous results (Figures 3A–C). To investigate the effect of IDO1 on AE progression, we extracted RNA from the liver cysts of infected mice and measured the levels of *14-3-3* and *II/3-10*. The expression of *14-3-3* and *II/3-10* in the cysts of WT mice was greater than that in the cysts of IDO1-KO mice (Figure 4A), confirming that IDO1 can promote AE progression, which is consistent with previous research [3]. Next, we evaluated the formation of NETs by detecting the levels of MPO–DNA complexes and cfDNA, which were significantly greater in WT mice than in IDO1-KO mice (Figures 4B, C). These data indicate that IDO1 may have an effect on the formation of NETs.

To further validate our conclusions, we isolated neutrophils from WT and IDO1-KO uninfected mice and cocultured them with PMA or cyst fluid. Then, we used electron microscopy and staining with anti-*CitH3* and anti-*NE* antibodies to detect the formation of NETs. Immunofluorescence analysis revealed a significant increase in the levels of *CitH3* and *NE* in mice after PMA stimulation. Moreover, the levels of *CitH3* and *NE* in IDO1-KO mice were notably lower than those in WT mice (Figure 4D–F). The data presented above demonstrated that IDO1 can promote the formation of NETs.

### The IDO1-NF-κB-IL-8 signalling axis promotes the formation of NETs

**Figure 5:**
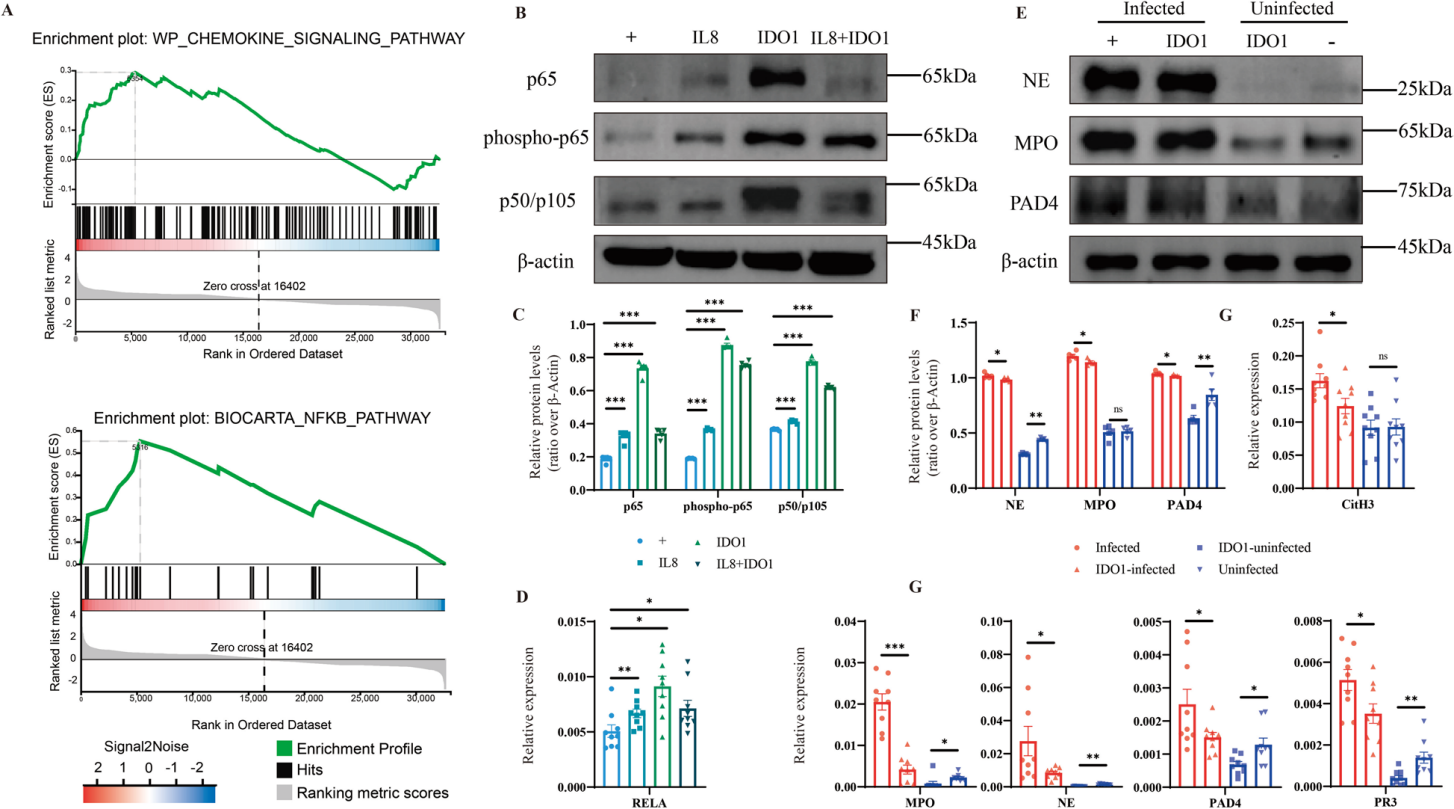
**IDO1 promotes the formation of NETs by influencing the activation of the NF-κB pathway.** **A** Gene set enrichment analysis of the NF-κB pathway and chemokine signalling pathway; **B** Immunoblot analysis of p65, phospho-p65, and p50/105 protein expression following stimulation with IDO1 and IL-8 inhibitors; **C** Bar chart for grayscale analysis of p65, phospho-p65, and p50/105 proteins via ImageJ software; **D** mRNA transcription levels of REL following stimulation with IDO1 and IL-8 inhibitors; **E** Immunoblot analysis of *MPO*, *PAD4* and *NE* proteins following IDO1 inhibitor stimulation; **F** Bar chart for grayscale analysis of *MPO*, *PAD4* and *NE* proteins via ImageJ software; **G** mRNA transcription levels of NET-related molecules (*MPO*, *PAD4*, *PR3*, *NE* and *CitH3*) following IDO1 inhibitor stimulation. “ + ” represents *E. multilocularis* stimulation, with “-” indicating no stimulation. The data are presented relative to the GAPDH values and are presented as the mean ± SEM; ns, not significant; *P < 0.05; **P < 0.01; ***P < 0.001.

Afterward, we studied the specific mechanism by which IDO1 regulates the formation of NETs. GSEA revealed that in the IDO1-KO group, the NF-κB and chemokine signalling pathways were activated (Figure 5A). To further validate our findings, we isolated neutrophils from WT uninfected mice and cocultured them with an IDO1 inhibitor, an IL8 inhibitor, or cyst fluid. We found that after the addition of the IDO1 inhibitor, the levels of p65, phospho-p65, p50/105, and REL increased. These results showed that the NF-κB signalling pathway was activated during *E. multilocularis* infection, but IDO1 inhibited the activation of NF-κB. Furthermore, although we suppressed the expression of IL-8, the level of NF-κB increased; moreover, the ability of IDO1 to induce IL-8 expression surpassed that of IL-8 inhibitors to suppress IL-8 expression, ultimately resulting in an increase in NF-κB levels. We speculated that IDO1 may influence the activation of NF-kB by increasing the levels of IL-8 (Figures 5B–D). Finally, we concluded that the formation of NETs is related to the IDO1–NF-κB–IL-8 signalling axis (Figures 5E–G).

## Discussion

Innate immune cells, including neutrophils and macrophages, play crucial roles in combating parasitic infections. Neutrophils, the first immune cells recruited to the site of infection, play an important role in mitigating the burden of early parasitic infections [36]. According to reports, parasites such as *Taenia solium* [37], *Trichomonas vaginalis* [38], and *Entamoeba histolytica* trophozoites [39] can induce an oxidative burst in neutrophils. In addition, *Leishmania braziliensis* is an important factor in stimulating neutrophil degranulation [40]. Some small parasites, such as *Trypanosoma cruzi* [41] and certain *Leishmania* species [42], are directly eliminated through phagocytosis by neutrophils; nevertheless, large parasites, such as worms, cannot be engulfed by neutrophils because of their size [43]. On this basis, neutrophils initiate another mechanism, NET formation, to respond to the invasion of some helminth parasites; NETs can capture worms due to their stickiness, thereby preventing further spread or development [44]. However, the role of neutrophils in *E. multilocularis* infection is not fully understood.

In this study, we observed a significant increase in the number of CD45 + CD11b + Ly6G + cells (neutrophils) after *E. multilocularis* infection. According to previous reports, the levels of NET-specific markers, such as free DNA and *MPO,* in patients with coronavirus disease 2019 (COVID-19) are closely related to the number of neutrophils [45]. Our findings confirmed this point: after *E. multilocularis* infection, the levels of cfDNA and MPO–DNA complexes and the expression levels of NET-related molecules, including *MPO*, *PAD4*, *PR3*, *CitH3*, and *NE*, were increased. Moreover, the increase in cfDNA and MPO–DNA complex levels suggests their potential as indicators for AE detection.

SEM analysis revealed that the cyst fluid of *E. multilocularis* can induce the formation of NETs in vitro, and these NET-like structures were confirmed via fluorescence confocal microscopy analysis. These results confirmed that *E. multilocularis* can significantly induce the formation of NETs, which is consistent with findings from studies on *L. amazonensis*, *Eimeria bovis* and *T. gondii* [46–48]. The NET formation process is accompanied by the release of *PR3*, histones, and granule proteins such as *NE* and *MPO* [49]. *PAD4*, an enzyme that converts arginine in histones to citrulline, plays a crucial role in the formation of NETs [50]. Consistent with reports of NETs induced by other parasites [14, 42], colocalization analysis revealed that *PAD4*, *PR3*, *MPO*, *CitH3*, and *NE* coexist in *E. multilocularis*-induced NETs, confirming the molecular characteristics of NETs.

According to previous studies, IDO1 inhibits T-cell activation by preventing the maturation of dendritic cells (DCs), which further promotes immune suppression and AE progression [23]. Similarly, MDSCs inhibit T-cell function in an IDO1-dependent manner in vitro, thus promoting the progression of AE [3]. The reduction in the expression levels of *14-3-3* and *II/3-10* in IDO1-KO mice observed in this study further suggests that IDO1 can promote the progression of AE. In addition, the antigens of *E. granulosus* can induce immune tolerance and facilitate infection by upregulating the expression of IDO1 [51]. The elevated IDO expression induced by *Leishmania* infection attenuates the host immune response and enhances the survival of *Leishmania* [52]. The antiparasitic mechanism of *T. gondii* during in vivo infection may also be associated with the inhibition of increased IDO activity [53].

Research has demonstrated that IDO1 enhances the recruitment of neutrophils into the abdominal cavity of mice during bacterial peritonitis and sepsis induced by cecal ligation and perforation [54]. During bovine pregnancy, the upregulation of IDO1 expression is essential for neutrophil chemotaxis [55]. We found that after *E. multilocularis* infection, the number of neutrophils in IDO1-KO mice was lower than that in WT mice, indicating that IDO1 plays a role in promoting the production of neutrophils; furthermore, IDO1 may facilitate the recruitment of neutrophils to the infected site in the mouse liver and may influence the differentiation of these neutrophils. Interestingly, the expression levels of *CitH3*, *NE*, *MPO*, *PR3*, and *PAD4* in IDO1-KO mice after infection were lower than those in WT mice; the same was true for the levels of free DNA and MPO–DNA complexes. SEM analysis also revealed that the NETs that formed in IDO1-KO mice after cyst fluid stimulation were more “loose” than those in WT mice. These data indicate that IDO1 may promote the formation of NETs. Even more interestingly, after excluding the influence of pathogens, we found that the levels of *NE* and *CitH3* in IDO1-KO mice were still lower than those in WT mice. This once again demonstrates that IDO1 has the capacity to influence the formation of NETs.

NF-κB is crucial for proper immune system function and serves as a key factor regulating the safety of immune responses at different stages [56]. CXCL8/IL-8, as an ELR + inflammatory chemokine, not only serves as an inducer for neutrophils but also stimulates the directed chemotaxis of white blood cells toward the site of inflammation [57]. Our research revealed that while *E. multilocularis* infection activates the NF-κB signalling pathway, IDO1 inhibits its activation. Interestingly, IDO1 can increase the expression of IL-8, which in turn activates NF-κB; however, our research indicated that the capacity of IDO1 to inhibit NF-kB significantly surpasses the ability of IL-8 to activate NF-kB. Additionally, IDO1 induces the expression of IL-8, and increased IL-8 expression recruits and activates more neutrophils, resulting in elevated levels of NETs. Thus, further research is necessary to investigate the intricate relationships among IDO1, NF-kB, and IL8 in the future. In addition, although our results indicate that IDO1 can regulate NF-κB and IL-8, the influence of aryl hydrocarbon receptor (AHR) [58] and proinflammatory factors, such as interferon-gamma (IFN-γ) [59], cannot be overlooked. Therefore, further research is necessary to elucidate the specific mechanisms by which IDO1 regulates NF-κB and IL-8.

Notably, the absence of IDO1 may activate various compensatory mechanisms to maintain homeostasis in the body. In liver fibrosis and cirrhosis, the absence of IDO1 results in a compensatory increase in tryptophan 2,3-dioxygenase (TDO) [60]. During *T. gondii* infection, the loss of IDO1 is compensated for by the upregulation of guanylate binding protein 1 (GBP1) and nitric oxide synthase 2 (NOS2) expression [61]. However, in studies on feto-maternal tolerance, the TDO pathway is not the primary mechanism that compensates for IDO1 deficiency [62]; in the epididymal heads of IDO1-deficient animals, the lack of IDO was not compensated for by other tryptophan-degrading enzymes [63]. In this study, we hypothesize that the absence of IDO1 following *E. multilocularis* infection does not involve compensatory mechanisms or that compensatory mechanisms, such as the TDO pathway, do not predominate. However, this requires further verification in future research. In summary, this study revealed that *E. multilocularis* can induce the formation of NETs and that IDO1 can increase the production of neutrophils during *E. multilocularis* infection. Moreover, the IDO1‒NF-κB‒IL-8 signalling axis regulates the formation of NETs, and IDO1 may inhibit the progression of AE by regulating the formation of NETs. These data may be conducive to understanding the pathogenesis of *E. multilocularis* and offer new insights for the prevention and treatment of AE.

## Data Availability

All the data are available in the figures, tables and figshare database [64].
